# Membrane-Pore Forming Characteristics of the *Bordetella pertussis* CyaA-Hemolysin Domain

**DOI:** 10.3390/toxins7051486

**Published:** 2015-04-30

**Authors:** Chattip Kurehong, Chalermpol Kanchanawarin, Busaba Powthongchin, Gerd Katzenmeier, Chanan Angsuthanasombat

**Affiliations:** 1 Bacterial Protein Toxin Research Cluster, Institute of Molecular Biosciences, Mahidol University, Salaya Campus, Nakornpathom 73170, Thailand; E-Mails: g5036578@student.mahidol.ac.th (C.K.); katzenmeier.ger@mahidol.ac.th (G.K.); 2 Laboratory of Theoretical and Computational Biophysics, Department of Physics, Faculty of Science, Kasetsart University, Bangkok 10900, Thailand; E-Mail: fscicpk@ku.ac.th; 3 Department of Biopharmacy, Faculty of Pharmacy, Silpakorn University, Nakornpathom 73000, Thailand; E-Mail: p.busaba.su@gmail.com; 4 Laboratory of Molecular Biophysics and Structural Biochemistry, Biophysics Institute for Research and Development (BIRD), Bangkok 10160, Thailand

**Keywords:** CyaA-Hly, *Bordetella pertussis*, cooperative action, hemolytic activity, oligomeric pores, single channels

## Abstract

Previously, the 126-kDa *Bordetella pertussis* CyaA pore-forming/hemolysin (CyaA-Hly) domain was shown to retain its hemolytic activity causing lysis of susceptible erythrocytes. Here, we have succeeded in producing, at large quantity and high purity, the His-tagged CyaA-Hly domain over-expressed in *Escherichia coli* as a soluble hemolytically-active form. Quantitative assays of hemolysis against sheep erythrocytes revealed that the purified CyaA-Hly domain could function cooperatively by forming an oligomeric pore in the target cell membrane with a Hill coefficient of ~3. When the CyaA-Hly toxin was incorporated into planar lipid bilayers (PLBs) under symmetrical conditions at 1.0 M KCl, 10 mM HEPES buffer (pH 7.4), it produced a clearly resolved single channel with a maximum conductance of ~35 pS. PLB results also revealed that the CyaA-Hly induced channel was unidirectional and opened more frequently at higher negative membrane potentials. Altogether, our results first provide more insights into pore-forming characteristics of the CyaA-Hly domain as being the major pore-forming determinant of which the ability to induce such ion channels in receptor-free membranes could account for its cooperative hemolytic action on the target erythrocytes.

## 1. Introduction

**Figure 1:**
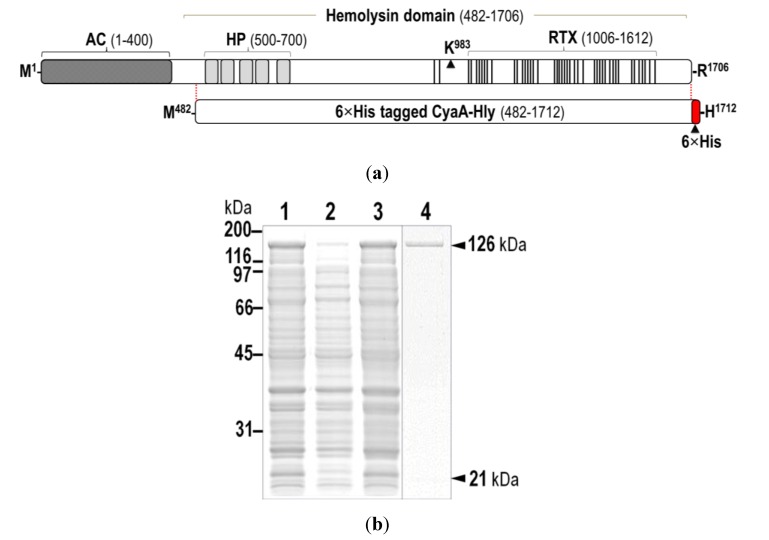

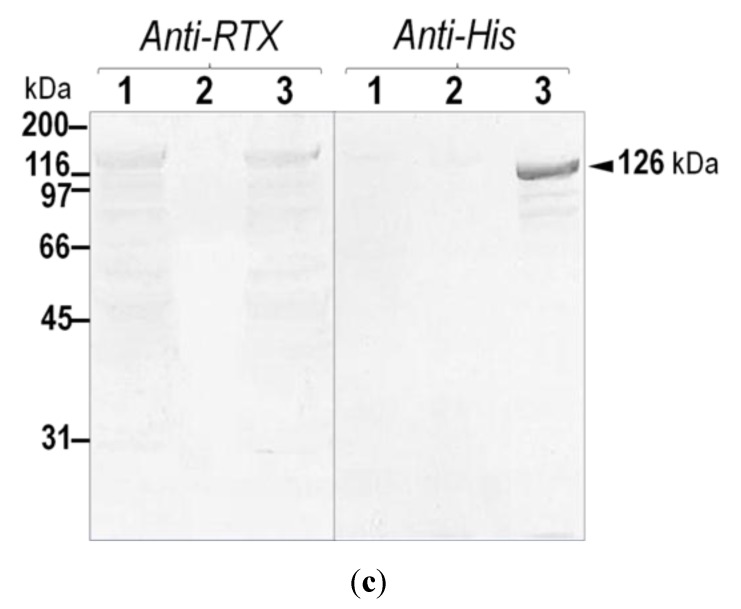
(**a**) (*Above*) Schematic diagram of CyaA showing adenylate cyclase (AC) and hemolysin (Hly) domains. Five putative helices, α1–α5, in the hydrophobic region (HP, residues 500–700) are represented by gray blocks. The palmitoylation site is indicated by Lys^983^, whereas the repeat in toxin (RTX) region (residues 1006–1612) is represented by multiple lines, with each line corresponding to a single nonapeptide repeat (X-U-X-Gly-Gly-X-Gly-X-Asp). (*Below*) Diagram of 6×His-tagged CyaA-Hly showing residues 482–1706 with the joining 6×His residues of 1707–1712; (**b**) SDS-PAGE (12% gel) stained with Coomassie Brilliant Blue of lysates extracted from *E. coli* (~10^6^ cells) harboring: lane 1, pCyaAC-PF with IPTG induction; lanes 2 and 3, pCyaAC-PF/H_6_ without and with IPTG induction, respectively. Lane 4 represents the Ni-NTA purified 6×His-tagged CyaA-Hly toxin. Protein bands of CyaA-Hly (~126 kDa) and its activator CyaC-acyltransferase (~21-kDa) are arrowed; (**c**) Western blot analyses of the corresponding gels from (b) incubated with anti-RTX (left panel) or anti-6×His epitope tag (right panel) antibodies, showing the reacted bands of 126-kDa CyaA-Hly as arrowed.

The bacterium *Bordetella pertussis* causes human whooping cough which has re-emerged and continued to be a major health problem worldwide, possibly due to the waning of vaccine-induced immunity and/or pathogen adaptation [1]. The 1706-amino acid adenylate cyclase-hemolysin toxin (also known as CyaA, see Figure 1a) secreted from *B. pertussis* is an important colonization factor for respiratory tract infection, presumably by assisting the pathogen to battle the host immune response via toxin-induced cytotoxicity to human macrophages [2]. Therefore, CyaA could be one of promising targets for current developments of novel approaches for whooping cough treatment [3].

Upon binding to the α_M_β_2_ integrin receptor (known as CD11b/CD18) on human macrophages. CyaA can translocate its *N*-terminal enzymatic domain-adenylate cyclase (AC) into the cytosol [4]. This catalytic domain massively generates cAMP-a key signalling molecule that affects transcription of many inflammatory-associated genes in apoptotic pathways leading to the death of target host cells [5]. CyaA can also exhibit hemolytic activity through its *C*-terminal pore-forming or hemolysin (Hly) domain (~126 kDa) against sheep erythrocytes which lack the integrin receptor [6,7,8]. Although this toxin-mediated hemolysis has been shown to be independent from the catalytic AC domain [6,7,8], the molecular mechanism of pore formation by the Hly domain has not completely described yet.

CyaA belongs to the family of RTX (Repeat in ToXin) cytolysins that are secreted by a number of pathogenic Gram-negative bacteria such as *E. coli*, *Moraxella* spp. and *Actinobacillus* spp. [9,10,11]. The RTX toxins share Gly-Asp rich nonapeptide repeats (X-U-X-Gly-Gly-X-Gly-X-Asp, X for any amino acid and U for large hydrophobic residues) that fold into a stable β-roll structure upon exposure to extracellular calcium ions at concentrations in the millimolar range [12,13]. This β-roll motif could mediate receptor recognition by which variation in number and organization of the RTX repeats would cause differences in target cell specificity of the RTX cytolysins [14]. The adjoining hydrophobic portion (HP) which possesses a highly conserved transmembrane region could possibly serve as a pore-forming component [10,11,15]. In view of the fact that there is currently no three-dimensional structure of RTX cytolysins, the HP region of the CyaA-Hly domain was previously predicted to adopt five transmembrane helices, *i.e.*, α1_(500-522)_, α2_(529–550)_, α3_(570–593)_, α4_(602–627)_ and α5_(678–698)_ [16]. In addition, our mutagenesis studies of the CyaA-Hly domain suggested that pore formation in the target cell membrane involves an insertion of the putative α2-loop-α3 hairpin [16]. We have further shown that polarity and/or charges at Glu^570^ and Glu^581^ of the α3 are important for hemolytic activity of CyaA-Hly [17]. Nevertheless, a more detailed understanding for structural basis of membrane-pore formation of the CyaA-Hly domain remains to be investigated.

In the present study, we have utilized a combined approach of hemolysis and ion-channel assays to provide further insights into membrane-pore-forming characteristics of the CyaA-Hly domain. Our results demonstrate that the CyaA-Hly domain is the major pore-forming determinant that forms oligomeric lytic pores on susceptible erythrocyte membranes as well as single channels on planar lipid bilayers.

## 2. Results and Discussion

### 2.1. Verification of the Expressed His-Tagged CyaA-Hly Domain

Previously, we have over-expressed the 126-kDa CyaA-hemolysin domain (CyaA-Hly) in *E. coli* together with its 21-kDa activator (*i.e*., CyaC-acyltransferase) from the pCyaAC-PF plasmid [8]. Although this construct was able to produce a high-yield soluble form of the hemolytically-active CyaA-Hly toxin, a large quantity and high quality of the pure target toxin could not be achieved via either anion-exchange combined with size-exclusion or antibody-affinity chromatography (data not shown). Here, we therefore re-constructed the recombinant plasmid, pCyaAC-PF/H_6_ (see Figure S1), encoding the CyaA-Hly domain fused at the *C*-terminus with 6×His-tag which was then exploited to enable an efficient one-step purification from crude lysates via immobilized metal affinity chromatography (IMAC, see data below).

Upon IPTG-induced expression, the 126-kDa His-tagged CyaA-Hly domain (Figure 1a) was produced as a soluble protein in *E. coli* at levels comparable to the untagged CyaA-Hly toxin (Figure 1b). The results in Figure 1c demonstrate that the His-tagged CyaA-Hly domain cross-reacted with anti-RTX and anti-6×His epitope tag antibodies, confirming its RTX identity as well as the presence of His-tag. Prior to toxin purification, *E. coli* crude lysate containing His-tagged CyaA-Hly was tested against sheep erythrocytes to ensure that the expressed fusion product retains its full functionality. The data revealed that the lysate with the His-tagged CyaA-Hly toxin exhibited high hemolytic activity comparable to the untagged toxin (66.0% ± 3.0% *versus* 63.0% ± 3.0%, see Table 1). Purification of the His-tagged CyaA-Hly toxin from lysate supernatant was accomplished via IMAC. Herein, a protein band with >95% purity of the 126-kDa His-tagged toxin bound to Ni-NTA was successfully recovered by a stepwise elution with 75 mM imidazole (Figure 1b, *lane 4*). It is worth mentioning that 2 mM CaCl_2_ plus certain protease inhibitors (*i.e.*, phenylmethylsulfonylfluoride, PMSF, and 1,10-phenanthroline, PNT) are required for toxin preparation (see Experimental Section 3.2 for details). These optimized conditions allowed us to obtain adequate amounts of the purified CyaA-Hly toxin (15–20 mg/L of culture) for further functional characterization.

**Table 1:** Hemolytic activity of CyaA-Hly toxins.

Toxin	Hemolytic activity ^b^ (%) ± SEM
CyaA-Hly cell lysate ^a^	63.0 ± 3.0
6×His-tagged CyaA-Hly cell lysate ^a^	66.0 ± 3.0

^a^ Soluble protein fraction of *E. coli* cell lysate (1 mg) containing ~10 μg CyaA-Hly toxins were incubated with sheep erythrocytes (5 × 10^8^ cells) in a total volume of 1 mL at 37 °C for 6 h; ^b^ Percent hemolysis was calculated as described in material and methods. The same amount of total proteins in the soluble fraction of *E. coli* lysate containing the pET17b vector gave <1% hemolysis (a negative control). The values were averaged from three independent experiments where each was performed in triplicates

### 2.2. Hemolytic Characteristics of the Soluble His-Tagged CyaA-Hly Toxin

**Figure 2:**
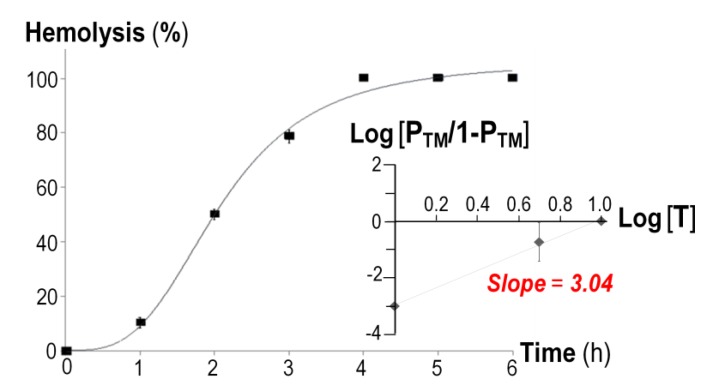
Time-course analysis of hemolytic activity of the purified His-tagged CyaA-Hly toxin (10 μg/mL) against sheep erythrocytes (5 × 10^8^ cells/mL). *Inset*, the plot of fractional hemolytic activity at 2 h, Y (P_TM_/1 − P_TM_), *versus* toxin concentration, [T]. Error bars indicate SEM from two independent experiments where each toxin concentration was done in triplicate.

The time course of *in vitro* hemolysis induced by the Ni-NTA purified CyaA-Hly toxin revealed a sigmoidal relationship (Figure 2), suggesting that leakage of the sheep erythrocyte membrane would require a lag period for toxin oligomerization since sheep erythrocytes were possibly not fully permeabilized until the toxin monomers had cooperatively formed a functional oligomeric pore which could initiate the colloid-osmotic hemolysis. In additional experiments, when the leakage of sheep erythrocytes was evaluated at different toxin concentrations, the concentration-versus-hemolytic activity profile evidently revealed positive cooperativity for oligomerization of the toxin monomer as indicated by a Hill coefficient (*n*) of 3.04 (Figure 2, *inset*) which is consistent with the previously reported *n* value for the 177-kDa full-length CyaA toxin [18]. Thus, hemolysis induced by the 126-kDa CyaA-Hly domain would require more than one molecule, conceivably at least three CyaA-Hly monomers, to form a functional trimeric pore structure in the erythrocyte membrane. This notion would be in good agreement with previous observations of undefined oligomers of the full-length CyaA toxin in the membrane of toxin-treated sheep erythrocytes [19]. However, the precise stoichimetry of CyaA-Hly pore assembly leading to hemolysis of the target cells still requires further investigation, while oligomeric pore structures of several hemolysins have been resolved by X-ray crystallography, including a homo-heptameric β-barrel of both *Staphylococcus aureus* α-hemolysin [20] and *Vibrio cholerae* cytolysin [21].

### 2.3. Ion-Channel Characteristics Formed by His-Tagged CyaA-Hly

**Figure 3:**
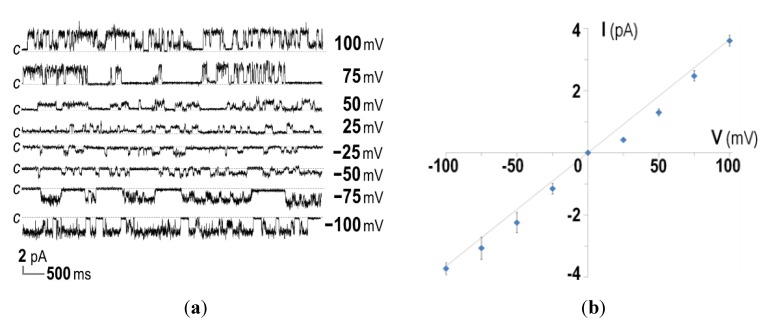

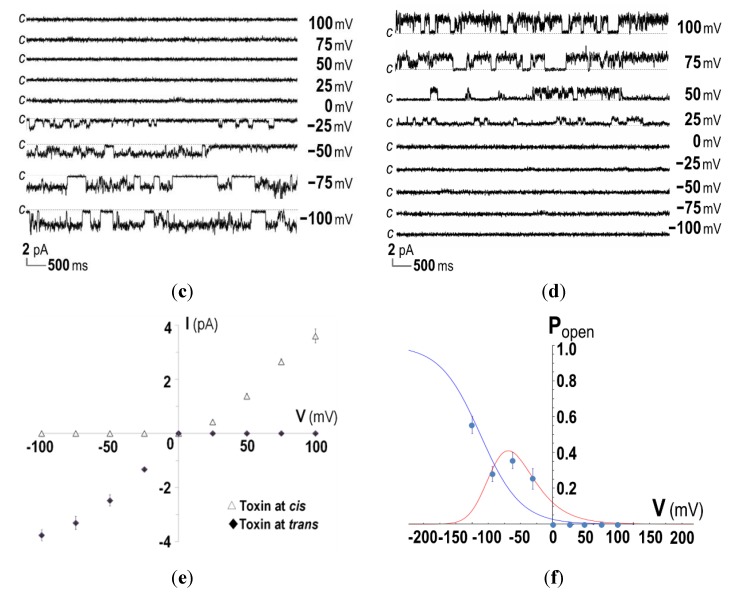
Ion-channel properties of CyaA-Hly on DiPhyPC (1,2-diphytanoyl-*sn*-glycero-3-phosphocholine) bilayers. (**a**) Current traces, I (pA) *versus* time (s), after incorporation of the purified CyaA-Hly toxin (1 μg/mL) into both chambers of PLBs in 1.0 M KCl, 10 mM HEPES buffer (pH 7.4). Applied voltages are indicated on the right side of each trace. The closed stage levels of the channel is denoted by the letter *c*; (**b**) The I-V curves of the PLB systems performed in the symmetrical conditions (1.0 M *cis*: 1.0 M *trans*); (**c**) Current traces, I (pA) *versus* time (s), after incorporation of CyaA-Hly (1 μg/mL) into either *trans* or (**d**) *cis* chamber of PLBs in 1.0 M KCl, 10 mM HEPES buffer (pH 7.4); (**e**) The I-V curves of the CyaA-Hly channel on DiPhyPC bilayers performed in 1.0 M KCl, 10 mM HEPES buffer (pH 7.4) in the presence of toxin in either *cis* or *trans* chamber; (**f**) Open probability, *P_open_* = *t_open_*/(*t_open_* + *t_closed_*), of CyaA-Hly channels determined from the PLB current traces in the presence of toxins in either *cis* or *trans* side at various membrane potentials and its theoretical fits calculated from the Boltzmann distribution, *P_open_* = 1/[1 + *e^(V−V')F/RT^*], where *V'* = −90 mV is the potential at a half maximum probability. Blue and red lines represent single and multiple modes of the toxin-induced channels, respectively.

Further attempts were made to biophysically characterize the channel-forming activity of the His-tagged CyaA-Hly domain by incorporation into planar lipid bilayers (PLBs). It was found that 15–20 min after addition of the purified His-tagged toxin under symmetrical ionic conditions (at 1 mM KCl, 10 mM HEPES, pH 7.4), the CyaA-Hly domain was able to induce single channel currents at voltages between −100 and +100 mV, as shown by representative current traces in Figure 3a. Considering the I-V curve for the toxin added into both *cis* and *trans* chambers, it can be seen that the CyaA-Hly channel could open and conduct ions at a maximum conductance of ~35 pS (Figure 3b). This conductance value is comparable to data previously reported for the 177-kDa full-length CyaA toxin [22], suggesting the CyaA-Hly domain plays a major role in channel formation of CyaA. It should be noted that several distinct sub-conductance levels were frequently detected for all tested voltages. These observations suggested a multimeric mode of toxin incorporation into the lipid bilayer. As was recently observed for a number of toxin-induced channels [23,24,25], the channels induced by CyaA-Hly most likely undergo conformational rearrangements during channel gating. Although currently a possible gating mechanism for the channels induced by CyaA-Hly and/or CyaA toxins has not been demonstrated unambiguously, our PLB experiments suggest that the CyaA-Hly channel could exhibit closed and open functional states.

It is also interesting to note that, when the CyaA-Hly toxin was added into the *trans* chamber, the toxin-induced channel could open only when negative potentials were applied to the *cis* chamber (*i.e*., *trans* was more positive) and it closed when the sign of the potential was reversed (see Figure 3c,e). In contrast when the toxin was added into the *cis* chamber, a positive potential was required to make it open and conduct ions and it closed otherwise (see Figure 3d,e). If the toxin inserts into the lipid bilayer only in one specific orientation, the channel induced by CyaA-Hly would open only when it experiences a negative membrane potential across its inserting direction, *i.e.*, polarity dependent. This polarity-dependent feature has also been observed in *E. coli* HlyA toxin-induced channels [26,27]. Thus, membrane potentials mimicking those of the cell would be necessary for the CyaA-Hly channel to function.

Data for determination of the channel open probability as illustrated in Figure 3f reveal that the channel induced by CyaA-Hly is voltage dependent, as it opens more frequently at higher negative membrane potentials with a ~50% open probability at about −90 mV. Such negative membrane potentials are generally found in mammalian cells (ranging from −10 to −90 mV) and these voltage- and polarity-dependent characteristics of the CyaA-Hly channel may suggest that the channel would open when it inserts into the target cell. In this case, the ion conduction of this toxin-induced channel came from the flow of K^+^ ions (and possibly Cl^−^ ions in the opposite direction) under the influence of the electric field across the PLB membrane. Further investigation for more details with different kinds of cations and anions under either symmetric or asymmetric conditions is of great interest since this PLB study would help us to understand which ions really participate in such CyaA-Hly channel conduction.

## 3. Experimental Section

### 3.1. Construction of Recombinant Plasmid with His-Tagged Fusion

A C-terminal 6×His tag was incorporated into the pCyaAC-PF plasmid which was previously used for over-expression of the soluble CyaA-Hly domain in *E. coli* [8]. The 670-bp *Xma*I-*Hind*III segment located at the 3'-end of the CyaA-Hly toxin gene was amplified with an added sequence encoding 6×His tag. The 688-bp PCR product was re-ligated into the pCyaAC-PF plasmid to replace the original sequence, giving pCyaAC-PF/H_6_ (7569 bp) that encodes the His-tagged CyaA-Hly domain (see Figure S1). The plasmid was transformed into *E. coli* strain BL21(DE3)pLysS and the sequence of the manipulated gene segment was verified by DNA sequencing.

### 3.2. Protein Expression

*E. coli* recombinant cells were cultured at 30 °C in Terrific Broth supplemented with ampicillin (100 μg/mL) and chloramphenicol (34 μg/mL). Toxin expression was induced by addition of 0.1 mM IPTG and further incubation for 4 h. After harvesting cells (6000× *g*, 4 °C, 10 min), the pellet was resuspended in 50 mM HEPES buffer (pH 7.4) containing 2 mM CaCl_2_ and 1.0 mM protease inhibitors (PMSF and PNT) and subsequently disrupted in a French Pressure Cell (10,000 psi). After centrifugation (13,000× *g*, 4 °C, 15 min), the lysate supernatant was analyzed by SDS–PAGE. The amount of CyaA-Hly in the supernatant of whole cell lysate was ~1% as estimated by visual comparison of the 126-kDa band intensity with the standard markers using a gel densitometer.

### 3.3. Western Blot Analysis

Protein samples separated on SDS–PAGE were transferred to a nitrocellulose membrane blocked with 5% skim milk-PBS (120 mM NaCl, 16 mM Na_2_HPO_4_, 4 mM NaH_2_PO_4_, pH 7.4). The blotted proteins were probed with rabbit anti-RTX antiserum (1:40,000 dilution) which was raised against the 100-kDa purified CyaA-RTX fragment as described previously [13]. Immune-complexes were detected with alkaline phosphatase (AP)-conjugated goat anti-rabbit IgG antibodies (Pierce, Rockford, IL, USA) at 1:7000 dilution and visualized by incubation with BCIP/NBT (5-bromo-4-chloro-3-indolyl phosphate/nitroblue tetrazolium). The presence of the 6×His tag was confirmed by probing with AP-conjugated anti-His (*C*-term) antibodies (Invitrogen, Waltham, MA, USA) at 1:2500 dilution and BCIP/NBT color detection.

### 3.4. Protein Purification

Purification of the His-tagged toxin was performed via IMAC using a Ni-NTA (nickel-nitrilotriacetic acid) column (5-mL HisTrap FF, GE Healthcare Bio-sciences, Buckinghamshire, UK). After injection of lysate supernatant (~25 mg), the column was washed with 20 mM imidazole (IMZ) in 50 mM HEPES buffer (pH 7.4) containing 2 mM CaCl_2_. Subsequently, the target protein was stepwise-eluted with 75 mM and 250 mM IMZ, respectively. Elution fractions containing the His-tagged toxin were pooled, analyzed by SDS–PAGE and desalted through a PD10 column (GE Healthcare Bio-sciences) prior to further analysis. Protein concentrations were determined by Bradford microassay (Bio-RAD, Hercules, CA, USA).

### 3.5. Hemolytic Activity Assay

*In vitro* hemolytic activity of the protein toxin against sheep erythrocytes was carried out as previously described [17]. The plot of fractional hemolytic activity, *Y* (*P*_TM_*/*1 − *P*_TM_), *versus* toxin concentration, [T], was fitted to the Hill equation, *P*_TM_*/*1 *− P*_TM_ *=* [T]*^n^*/*EC*_50_, where *P*_TM_ is the probability of finding toxin-membrane complex (T_n_M) to give a Hill coefficient (*n*) and an effective concentration (*EC*_50_). The coefficient *n* was obtained from the slope of the logarithmic Hill plot, log *Y* = *n* log [T] – log *EC*_50_ [23].

### 3.6. Planar Lipid Bilayers and Single Channel Analysis

PLBs were formed by painting 20 mg/mL of DiPhyPC (Avanti Polar Lipids, Alabaster, AL, USA) on a 250-µm aperture in a 1 mL-Delrin cup (Warner Instruments, Hamden, CT, USA), with a membrane capacitance value of ~250 pF. Incorporation of CyaA-Hly (at amounts of ~1 μg/mL prepared in 20 mM HEPES buffer, pH 7.4, 5 mM CaCl_2_) into PLBs was facilitated by stirring the protein-containing buffer (1 M KCl, 10 mM HEPES buffer, pH 7.4) in either *cis* or *trans* compartment while applying a 100-mV holding potential across the lipid bilayer. Single-channel currents were recorded with Geneclamp-500 amplifier (Axon Instruments, Sunnyvale, CA, USA). Signals, which were filtered at 10 kHz, were digitized with a PCI-6221 analogue-to-digital converter (National Instruments, Austin, TX, USA) using LabVIEW 7.1 software at a 50-kHz sampling frequency. Channel conductance was determined from the slope of current-voltage (I-V) relations plotted between the observed current steps and the corresponding applied voltage.

## Supplementary Materials

Supplementary materials can be accessed at: http://www.mdpi.com/2072-6651/7/5/1486/s1.

## Abbreviations

CyaA: adenylate cyclase-hemolysin toxin
DiPhyPC: 1,2-diphytanoyl-*sn*-glycero-3-phosphocholine
Ni-NTA: nickel-nitrilotriacetic acid
PLBs: planar lipid bilayers
PMSF: phenylmethylsulfonylfluoride
PNT: 1,10-phenanthroline
RTX: Repeat in ToXin
