# Supplementary material for: Membrane-Pore Forming Characteristics of the Bordetella pertussis CyaA-Hemolysin Domain
Source: Toxins (Basel). 2015 Apr 30;7(5):1486–96. doi: 10.3390/toxins7051486 (PMC4448159; doi:10.3390/toxins7051486)
Supplement: Supplementary file 1 [file toxins-07-01486-s001.pdf]

Supplementary Information

(**a**)

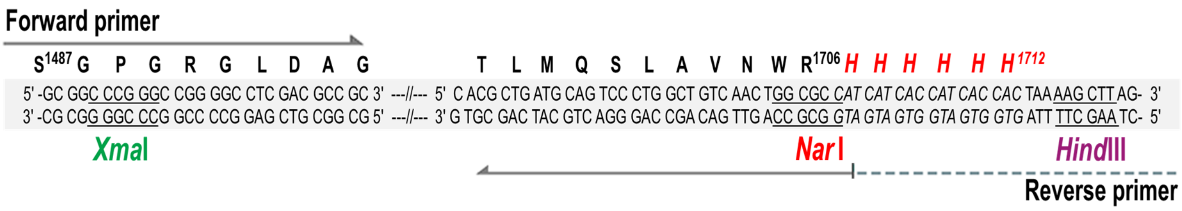

(**b**)

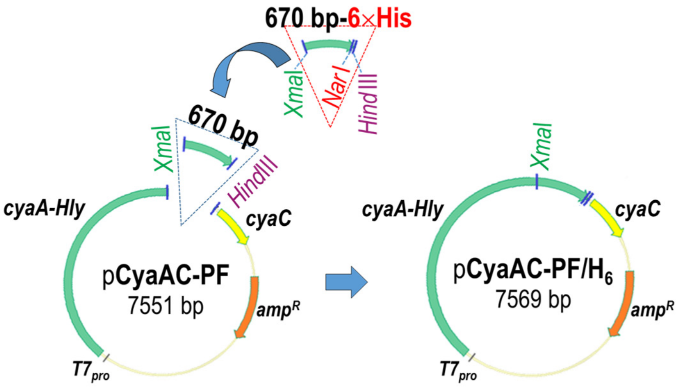

(**c**)

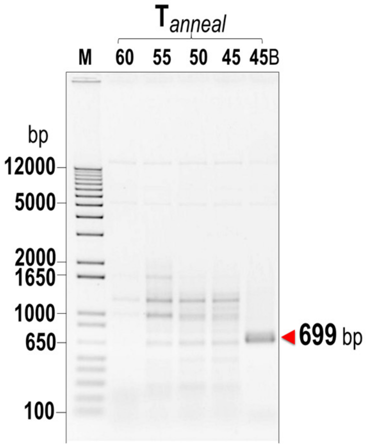

Figure S1. Construction of the recombinant pCyaAC-PF/H_6 plasmid with His-tagged fusion. (**a**) Two primers which were designed for amplification of the *Xma*I-*Hind*III fragment (670 bp) of the CyaA-Hly toxin gene. The forward primer contains *Xma*I site. The reverse primer contains 29-bp overhanging DNA sequences encoding 6× His tag and restriction sites for *Nar*I and *Hind*III; (**b**) The 7569-bp pCyaAC-PF/H_6 (*right*) was constructed from 7551-bp pCyaAC-PF (*left*); (**c**) Agarose gel electrophoresis of the PCR products amplified at annealing temperature ranging from 45–60 °C. The 699-bp expected PCR products (*arrowed*) was amplified at 45 °C in the presence of betaine enhancer.
